# Patient-reported health-related quality of life after a displaced intra-articular calcaneal fracture: a systematic review

**DOI:** 10.1186/s13017-015-0056-z

**Published:** 2015-12-30

**Authors:** G. Alexandridis, A. C. Gunning, L. P. H. Leenen

**Affiliations:** Department of Surgery, University Medical Center Utrecht, Suite: G04.228, Heidelberglaan 100, 3584 CX Utrecht, The Netherlands

**Keywords:** Displaced intra-articular calcaneal fracture, Systematic review, Quality of life, Long-term follow up, SF-36, EQ-5D

## Abstract

**Background:**

A displaced intra-articular calcaneal fracture (DIACF) is known for having a negative influence on the daily activities of patients. A health-related quality of life (HRQoL) outcome instrument is used to quantify the impact of DIACF. It seems that these studies used restrictive inclusion criteria and observe specific patient groups; consequently, an increased risk of bias that results in incorrect estimation of the impact. Therefore, we will systematically review the current literature.

**Materials and methods:**

A systematic search was performed in PubMed, Embase and Cochrane library. Inclusion criteria were studies reporting DIACF and HRQoL, measured with SF-36, SF-36v2, EQ-5D or EQ-6D. The identified articles were critically appraised for their relevance and validity. The overall risk of bias was determined. The studies with a low to medium risk of bias were used for data extraction.

**Results:**

32 articles were available for the critical appraisal. 13 articles had a medium risk of bias. All studies reported the SF-36 and two studies also reported the EQ-5D.

**Conclusions:**

This systematic review indicates that DIACF is a life-changing event for most patients. The HRQoL is substantially lower in comparison to the period before the trauma and to the general population, in particular the subdomains related to the physical domain are affected. In addition, this review reveals that the identified studies have a medium to high risk of bias. Consequently, it is challenging to make reliable and valid conclusions. Therefore, we provided recommendations to decrease the risk of bias in order to improve future research.

## Background

It is well-known among orthopedic healthcare providers that a displaced intra-articular calcaneal fracture (DIACF) has a negative influence on the daily activities of patients and affects their quality of life substantially [1, 2]. Moreover, this fracture has an adverse economic impact on society. Buckley et al. [1] showed that approximately 20 % of these patients do not return to work after 1 year.

A health-related quality of life (HRQoL) outcome instrument is used to quantify the impact of a DIACF on the daily activities of these patients [1, 3–8]. Policy makers use these HRQoL outcomes for economic assessments and to optimize the use of the scarce resources. Improving HRQoL might result in an increased number of patients who return to work, and consequently this will increase the cost-effectiveness of the management of patients with a DIACF.

In current literature studies were performed that use HRQoL as outcome. These studies demonstrated to have lower HRQoL scores in comparison to the general population [1, 7, 8]. However, these studies tend to use restrictive inclusion criteria and to observe specific patient groups [1, 6–8]. As a consequence, these studies are prone to provide results that seem too positive to us, and might underestimate the need for advancements of research to improve the daily lives of patients with a DIACF.

Previous systematic reviews have reported HRQoL on DIACF, but focused mainly on comparing treatments with outcomes such as complications, return to work rate, or the ability to wear certain shoes. As a result, these reviews have not included all the available original studies on HRQoL after a DIACF; subsequently, this may bias the HRQoL outcome after a DIACF [9–17].

Therefore, a thoroughly and explicit assessment of the validity of primary studies that use HRQoL as an outcome is required. With this knowledge, more insight in the disease specific progression could be established and the rehabilitation process could be improved.

This is the first systematic review, which focuses solely on the HRQoL in adult patients who sustained a traumatic DIACF. We will critically appraise the validity of current literature. As a result, we will present a comprehensive overview of the quality of these studies and provide a thorough update on the best available evidence of the HRQoL status in patients with a DIACF.

## Review

### Introduction

It is well-known among orthopedic healthcare providers that a displaced intra-articular calcaneal fracture (DIACF) has a negative influence on the daily activities of patients and affects their quality of life substantially [1, 2]. Moreover, this fracture has an adverse economic impact on society. Buckley et al. [1] showed that approximately 20 % of these patients do not return to work after 1 year.

A health-related quality of life (HRQoL) outcome instrument is used to quantify the impact of a DIACF on the daily activities of these patients [1, 3–8]. Policy makers use these HRQoL outcomes for economic assessments and to optimize the use of the scarce resources. Improving HRQoL might result in an increased number of patients who return to work, and consequently this will increase the cost-effectiveness of the management of patients with a DIACF.

In current literature studies were performed that use HRQoL as outcome. These studies demonstrated to have lower HRQoL scores in comparison to the general population [1, 7, 8]. However, these studies tend to use restrictive inclusion criteria and to observe specific patient groups [1, 6–8]. As a consequence, these studies are prone to provide results that seem too positive to us, and might underestimate the need for advancements of research to improve the daily lives of patients with a DIACF.

Previous systematic reviews have reported HRQoL on DIACF, but focused mainly on comparing treatments with outcomes such as complications, return to work rate, or the ability to wear certain shoes. As a result, these reviews have not included all the available original studies on HRQoL after a DIACF; subsequently, this may bias the HRQoL outcome after a DIACF [9–17].

Therefore, a thoroughly and explicit assessment of the validity of primary studies that use HRQoL as an outcome is required. With this knowledge, more insight in the disease specific progression could be established and the rehabilitation process could be improved.

This is the first systematic review, which focuses solely on the HRQoL in adult patients who sustained a traumatic DIACF. We will critically appraise the validity of current literature. As a result, we will present a comprehensive overview of the quality of these studies and provide a thorough update on the best available evidence of the HRQoL status in patients with a DIACF.

### Materials and methods

The Preferred Reporting Items for Systematic Reviews and Meta-Analyses (PRISMA) guideline was used to conduct this review [18].

#### Health-related quality of life (HRQoL)

HRQoL is the subjective evaluation of the aspects that affect a person’s health. These aspects are classified into the physical, psychological, and social domain. These domains can be further specified into subdomains. Pain and physical functioning are subdomains of the physical domain. The psychological domain is further specified in emotional and mental health. The quality of social contacts and behavior are subdomains of the social domain.

For the assessment of HRQoL, several instruments have been developed and validated, such as the Medical Outcomes Study 36-item Short Form Health Survey (SF-36) and the EuroQol 5D (EQ-5D) [19–22]. There is, to our knowledge, no validated patient reported outcome which specifically addresses patients with a DIACF or patients with an ankle or foot disorder. The literature states that SF-36 can be used as a valid instrument to measure HRQoL in these patients [3–5].

#### Search strategy

A computerized search was conducted on March 15, 2015 in the search engines PubMed, Cochrane library and Embase. The search syntax encompassed *calcaneus, fracture*, and the outcome instruments: *SF-36, SF-36v2, EQ-5D* and *EQ-6D (*including their respective synonyms) [19–22]. Table 1 contains an overview of the complete search syntax. We searched titles and abstracts. All articles, which met the search terms, were exported from the search engines to Reference Manager version 12.0.

**Table 1:** Search syntax per search engine. Search conducted on 15th March of 2015

PubMed:
(calcaneus[tiab] OR calcaneum[tiab] OR calcaneal[tiab] OR calcis[tiab] OR kalkaneus[tiab] OR (heel[tiab] AND bone[tiab]) OR fersenbein[tiab] OR hielbeen[tiab] OR hielbot[tiab] OR calcanean[tiab] OR calcanea[tiab]) AND (fracture[tiab] OR fractures[tiab] OR lesion[tiab] OR lesions[tiab] OR broken[tiab] OR fractured[tiab] OR splintered[tiab] OR displaced[tiab] OR displacement[tiab] OR discontinuity[tiab] OR discontinuities[tiab] OR gebroken[tiab] OR fracture[tiab] OR frakturen[tiab] OR fractuur[tiab] OR fracturen[tiab] OR fragmentation[tiab] OR fragmented[tiab] OR fragment[tiab] OR cleavage[tiab] OR cleavages[tiab]) AND (sf36[tiab] OR sf-36[tiab] OR (sf[tiab] AND 36[tiab]) OR (health[tiab] AND (survey[tiab] OR surveys[tiab] OR review[tiab] OR reviews[tiab] OR questionnaire[tiab] OR questionnaires[tiab])) OR (short[tiab] AND (form[tiab] OR form-36[tiab])) OR short-form[tiab] OR euroqol[tiab] OR euroqol-5[tiab] OR euroqol-6[tiab] OR eq-5d[tiab] OR eq-6d[tiab] OR (quality[tiab] AND life[tiab]) OR ((clinical[tiab] OR functional[tiab]) AND (assessment[tiab] OR score[tiab] OR scores[tiab] OR outcome[tiab] OR outcomes[tiab])))
Embase:
calcaneus:ab,ti OR calcaneum:ab,ti OR calcaneal:ab,ti OR calcis:ab,ti OR kalkaneus:ab,ti OR (heel:ab,ti AND bone:ab,ti) OR fersenbein:ab,ti OR hielbeen:ab,ti OR hielbot:ab,ti OR calcanean:ab,ti OR calcanea:ab,ti AND (fractures:ab,ti OR lesion:ab,ti OR lesions:ab,ti OR broken:ab,ti OR fractured:ab,ti OR splintered:ab,ti OR displaced:ab,ti OR displacement:ab,ti OR discontinuity:ab,ti OR discontinuities:ab,ti OR gebroken:ab,ti OR fracture:ab,ti OR frakturen:ab,ti OR fractuur:ab,ti OR fracturen:ab,ti OR fragmentation:ab,ti OR fragmented:ab,ti OR fragment:ab,ti OR cleavage:ab,ti OR cleavages:ab,ti) AND (sf36:ab,ti OR ‘sf 36’:ab,ti OR (sf:ab,ti AND 36:ab,ti) OR (health:ab,ti AND (survey:ab,ti OR surveys:ab,ti OR review:ab,ti OR reviews:ab,ti OR questionnaire:ab,ti OR questionnaires:ab,ti)) OR (short:ab,ti AND (form:ab,ti OR ‘form 36’:ab,ti)) OR ‘short form’:ab,ti OR euroqol:ab,ti OR ‘euroqol 5’:ab,ti OR ‘euroqol 6’:ab,ti OR ‘eq 5d’:ab,ti OR ‘eq 6d’:ab,ti OR (quality:ab,ti AND life:ab,ti) OR (clinical:ab,ti OR functional:ab,ti AND (assessment:ab,ti OR score:ab,ti OR scores:ab,ti OR outcome:ab,ti OR outcomes:ab,ti)))
Cochrane Library: searched in title, abstract and keywords
(calcaneus OR calcaneum OR calcaneal OR calcis OR kalkaneus OR (heel AND bone) OR fersenbein OR hielbeen OR hielbot OR calcanean OR calcanea) AND (fracture OR fractures OR lesion OR lesions OR broken OR fractured OR splintered OR displaced OR displacement OR discontinuity OR discontinuities OR gebroken OR fracture OR frakturen OR fractuur OR fracturen OR fragmentation OR fragmented OR fragment OR cleavage OR cleavages) AND (sf36 OR sf-36 OR (sf AND 36) OR (health AND (survey OR surveys OR review OR reviews OR questionnaire OR questionnaires)) OR (short AND (form OR form-36)) OR short-form OR euroqol OR euroqol-5 OR euroqol-6 OR eq-5d OR eq-6d OR (quality AND life) OR ((clinical OR functional) AND (assessment OR score OR scores OR outcome OR outcomes)))

#### Selection

Our aim was to analyze all articles evaluating the HRQoL in patients with DIACF; therefore, we have included all articles written in English, German, French or Dutch that assessed patients who had a primary treated DIACF and reported results of SF-36, SF-36v2, EQ-5D, or EQ-6D.

**Fig. 1:**
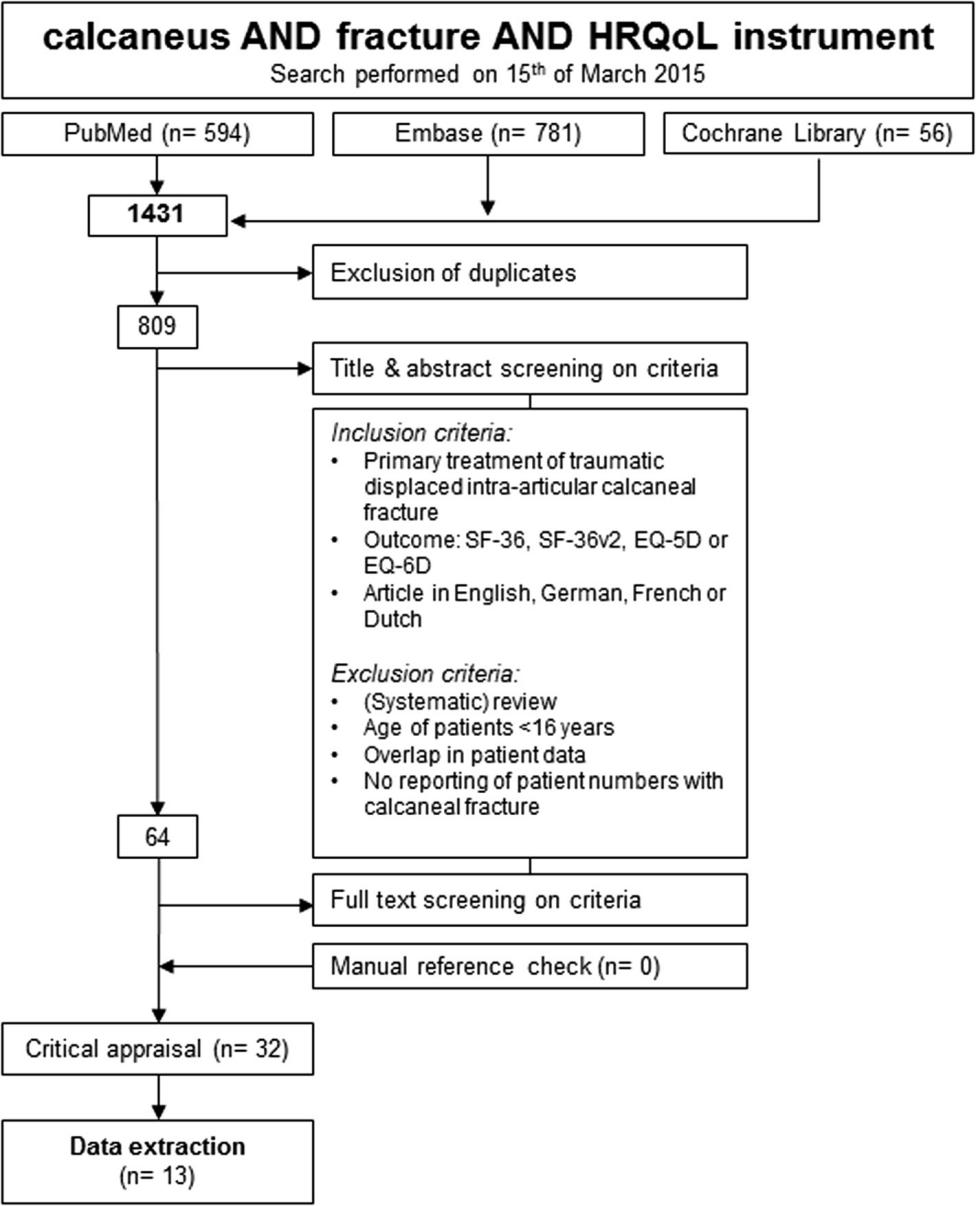
Flow-chart of search strategy and selection

At first, all duplicates were excluded. The titles and abstracts of the articles were screened by one reviewer (GA), based on the inclusion criteria (Fig. 1). We have excluded articles that assessed patients who were aged younger than 16 years. In addition, we excluded articles that are reviews. The full text of the remaining articles was screened by two reviewers (GA, ACG). From these articles and the identified systematic reviews, all references were screened using the same criteria. In case of overlapping patient data, the article with the highest number of patients was included.

#### Critical appraisal

Predefined criteria were used for the assessment of validity and relevance in the selected articles; these criteria are presented in Table 2. Relevance concerned the applicability of the study findings to adult patients who sustained a DIACF. The preference was to use calcaneal fracture classification to determine intra- versus extra-articular fractures. In case a calcaneal fracture classification was not reported, we used the population criteria whether there were patients with intra- and extra-articular calcaneal fractures included.

**Table 2:** Legend by Table 2: critical appraisal

Study design	+	Prospective study or randomized controlled trial	
	±	Retrospective study	
	-	Other, such as case report	
*Relevance*			
Domain	+	Studies including adult trauma patients with DIACF	
	±	Studies including adult trauma patients with other calcaneal fractures	
	-	Studies including adult trauma patients with other solitary non-calcaneal fractures	
Outcome	+	SF-36, SF-26v2, EQ-5D or EQ-6D	
	-	Other	
*Validity*			Points
Transparency of methodology	+	Clearly described methodology	-
	-	Not clearly described methodology or not reported	-
Characteristics of study population (age, classification of calcaneal fracture, length of follow-up, and associated injuries)	+	Clearly described study population characteristics and inclusion of all characteristics	-
	-	Not clearly described study population characteristics or missing description of characteristics	-
Minimal selection bias	+	All eligible patients included in the study population	+5
	±	Pre-selected patient groups or subgroups	+3
	-	Subjective selection of eligible patients or not reported	−3
Outcome assessment	+	Complete mean crude scores per subdomain in patient group, or crude scores per subdomain	+6
	±	Incomplete mean crude scores per subdomain, or mean cumulative scores or EQ-5D index scores	+4
	-	No subdivision of the mean total scores in the subdomains or cumulative scores	0
Lost to follow-up	+	<15 %	+4
	±	15 to 25 %	+2
	-	>25 % or not reported	0
Missing data	+	Description of patient characteristics who are lost to follow-up and how the missing data was handled	+1
	-	No description of patient characteristics who are lost to follow-up or how missing data was handled	0
Standardization of the conduction of the questionnaires	+	Standardized	+2
	-	Not standardized or not reported	0
			Total points
Overall risk of bias	+	Low risk of bias	14 to 18
	±	Medium risk of bias	10 to 13
	-	High risk of bias	−3 to 9

Validity assessment established the extent of selection and information bias. For the assessment of the risk of bias we used the criteria for a prognostic study design from the Centre for Evidence Based Medicine of the University of Oxford, and criteria used in previous systematic reviews, which addressed HRQoL [23–26]. Two reviewers (GA, ACG) appraised the identified articles separately. In case of a difference in the critical appraisal of the selected articles between the two reviewers (GA, ACG), consensus was reached through discussion or a third reviewer (LPHL) was asked.

We developed a scoring system to determine the overall methodological quality of the studies, due to the large heterogeneity in the designs of the studies. As presented in the literature, priority criteria which are likely to be essential for the methodological quality have the highest weight in our scoring system [26]. Description of population characteristics is important for the interpretation of the results; it is generally accepted that the type of fracture and the length of follow-up have a substantial influence on the outcome [27, 28]. Nevertheless, as described in the *Cochrane Handbook for Systematic Reviews of Interventions,* we did not value the transparency of methodology and reporting of population characteristics, because it is an uncertainty in the assessment of the risk of bias [29].

Eventually, we assigned points for each ‘validity’ criterion (Table 2 demonstrated the scores per criterion). Afterwards, we summed all these points per study (possible range −3 to 18 points). The total points of the studies were separated in three levels: high (−3 to 9 points), medium (10 to 13 points) or low risk of bias (14 to 18 points). Only articles with a low or medium risk of bias will be included in this systematic review and used for data extraction.

#### Data extraction

The SF-36 and SF-36v2 score ranges from 0 to 100 per subdomain; the higher the score the better the outcome [21, 22]. These instruments measure HRQoL across eight subdomains. The subdomain ‘physical functioning’ scores the performance of physical activities, ‘role-physical’ grades the limitations in daily activities as a result of physical health, and ‘bodily pain’ assesses the restrictions due to pain. ‘General health’ evaluates personal health, and ‘vitality’ measures energy and fatigue. ‘Social functioning’ is the subdomain score for interference due to emotional and physical problems with normal social activities. The ‘role-emotional’ subdomain measures the problems of daily activities as a result of emotional problems, and ‘mental health’ determines the psychological distress and well-being. These subdomains can be reduced to a ‘physical component summary’ (PCS) and a ‘mental component summary’ (MCS) score [21, 22].

The SF-36 and SF-36v2 have some differences in response choices, questions and in the calculation of the scores; the scores of the SF-36 and the SF-36v2 are comparable and can be combined [21, 22, 30].

The index score for EQ-5D ranges from below zero to one. A score of 1.00 is the highest possible score and indicates a patient who experiences no limitations in any of the subdomains, 0 indicates a HRQoL comparable to death, and a value below zero indicates a HRQoL worse than death [20].

We preferred to extract mean scores with standard deviation per subdomain. If these data were not available, we have calculated this data from the data presented in the studies. All data per group in the study were summed to calculate one overall mean score. In case we were not able to calculate mean scores from the presented data, we have contacted the authors by e-mail or telephone to ask for these data.

In order to present the development of the HRQoL through the years, we have reported the results in order of mean follow-up time. If possible, the data of the studies will be pooled to calculate the mean scores per subdomain. The homogeneity of the articles will be determined with eyeballing [31].

### Results

#### Search strategy & selection

The search resulted in 1431 articles. First of all, we have excluded the duplicates, as a result 810 articles remained. Title and abstract screening was performed using predefined inclusion criteria, 64 articles were left for full text and reference screening. No additional articles were identified during the reference check. Sixteen articles had overlap in patient data. Eventually, 32 articles were available for the critical appraisal [6–8, 27, 28, 32–58]. The flowchart is presented in Fig. 1.

#### Critical appraisal

An overview of the critical appraisal is presented in Table 3. Prior to critical appraisement, the authors of 24 articles were contacted for the necessary data [6–8, 28, 32–36, 39–41, 43–45, 47–52, 55, 58]. Only De Groot et al. [48] provided their study data, and gave permission to present their data in this review.

**Table 3:** Critical appraisal (see Table 2 for the legend)

		*Relevance*		*Validity*							
	Study design	Domain	Outcome	Transparency of methodology	Characteristics of population	Minimal selection bias	Outcome assessment	Lost to follow-up	Missing data	Standardization of conduction	Risk of Bias
Hildebrand (1996) [32]	RCS	+	+	+	-	-	-	+	NA	-	-
Heffernan (2000) [33]	RCS	+	+	-	-	+	±	+	-	-	±
Kennedy (2003) [34]	RCS	+	+	-	-	-	-	+	NA	-	-
Berry (2004) [35]	RCS	+	+	+	+	±	±	-	-	+	-
Van Tetering (2004) [7]	RCT	+	+	+	-	±	+	±	-	+	±
Westphal (2004) [8]	RCS	+	+	+	+	±	+	+	-	-	±
Herscovici (2005) [36]	RCS	±	+	+	+	±	-	±	-	+	-
Allmacher (2006) [27]	RCS	+	+	+	-	±	±	-	-	-	-
Robb (2007) [37]	RCS	+	+	+	-	-	+	±	-	+	-
Johal (2009) [38]	RCT	+	+	+	-	+	+	±	-	-	±
Rubino (2009) [28]	RCS	+	+	+	-	±	-	-	-	+	-
Wee (2009) [39]	PCS	+	+	+	+	±	-	-	+	-	-
DeWall (2010) [40]	RCS	+	+	+	-	+	±	-	-	-	-
Kinner (2010) [41]	PCSS	+	+	+	+	±	+	NR	-	+	±
Makki (2010) [6]	RCS	+	+	+	+	±	±	+	-	+	±
Hirschmüller (2011) [42]	RCS	±	+	+	+	-	+	+	-	-	-
Tomesen (2011) [43]	RCS	+	+	+	+	±	-	±	-	+	-
Woon (2011) [44]	PCS	+	+	+	-	±	-	NR	-	-	-
Beltran (2012) [45]	RCS	+	+	+	-	±	±	+	-	-	±
Brunner (2012) [46]	RCS	+	+	+	-	±	+	±	-	+	±
Ågren (2013) [47]	RCT	+	+	+	-	±	±	-	-	+	-
De Groot (2013) [48]	RCS	+	+	+	-	±	+	-	-	+	±
Demiralp (2013) [49]	RCS	±	+	+	-	-	-	NR	-	+	-
Kline (2013) [50]	RCS	+	+	+	-	±	-	-	-	+	-
Rammelt (2013) [51]	RCS	+	+	+	-	+	±	-	-	-	-
Tornetta (2013)[52]	PCS	±	+	+	-	-	±	NR	-	-	-
Buckley (2014) [53]	RCT	+	+	+	±	±	±	±	-	+	±
Chen (2014) [54]	RCS	+	+	+	±	±	-	NR	-	+	-
De Boer (2014) [55]	RCS	+	+	+	+	±	±	-	+	+	±
Griffin (2014) [56]	RCT	+	+	+	+	±	±	±	+	+	±
Persson (2014) [57]	RCS	±	+	+	+	±	±	-	-	+	-
Sanders (2014) [58]	PCS	+	+	+	+	±	±	-	+	+	±

*RCT* randomized controlled trial, *PCS* prospective cohort study, *PCSS* prospective cross sectional study, *RCS* retrospective cohort study, *NA* not applicable, *NR* not reported

Ten of the 32 articles have a prospective study design [7, 38, 39, 41, 44, 47, 52, 53, 56, 58]. Five articles did not entirely fulfill the criteria for relevance; these studies included non-displaced calcaneal fractures or other foot/ankle fractures [36, 42, 49, 52, 57]. The majority of the articles did not provide all the patient characteristics of the population they have studied [7, 27, 28, 32–34, 37, 38, 40, 44–54]. In the greater proportion of these studies, the associated injuries were not clearly described [7, 27, 28, 33, 34, 37, 38, 40, 42, 43, 47–51, 53, 54, 56–58]. Several studies did not report the classification of calcaneal fracture [7, 27, 32, 33, 48, 49, 52].

Four of the 32 articles have a minimal selection bias [33, 38, 40, 51]. The other studies excluded specific patient groups, such as patients with open fractures, concomitant injuries, primary or secondary subtalar arthrodesis, systemic comorbidities or bilateral fractures. Furthermore, some studies only included one specific patient group with DIACF, e.g. solely patients with open fractures or one specific treatment [45, 53].

The complete mean crude scores per subdomain were presented in seven [7, 8, 37, 38, 41, 42, 46]; the other articles provided cumulative scores or did not report all the scores per subdomain. Ten articles provided a ‘total’ of SF-36 scores, which represents the mean score of all the eight subdomains of the SF-36 [28, 32, 34, 36, 39, 43, 44, 49, 50, 54].

Seven articles had a follow-up rate above 85 % [6, 8, 32–34, 42, 45]. Five articles did not present any follow-up data [41, 44, 49, 52, 54].

The authors of four studies presented data of patients who were lost to follow up [39, 55, 56, 58].

In 13 studies the inclusion of patients and the collection of patient data were not standardized or this information was not described in the studies [8, 27, 32–34, 38–40, 42, 44, 45, 51–53].

After the critical appraisal, 13 articles were scored with a medium risk of bias and were selected for the data extraction, none of the articles had a low risk of bias [6–8, 33, 38, 41, 45, 46, 48, 53, 55, 56, 58].

#### Data extraction

The results of the data extraction are presented in Table 4.

**Table 4:** Study population characteristics and SF-36 outcome results of selected studies

	Questionnaire	Number of patients (n=)	Reported classification of calcaneal fracture type	Mean age in years (range)	Other characteristics of studied population	Treatment	Mean follow-up time in months (range)	SF-36 mean scores									
								Physical functioning	Role-physical	Bodily pain	General health	Vitality	Social functioning	Role-emotional	Mental health	Physical component summary	Mental component summary
Johal (2009) [38]	SF-36	41	Sanders	36 (18–61)	Exclusion of open fractures, aged >65 years, medical contraindications or intoxication abuses	ORIF	>12 (NR)	59	40	51	77	63	67	65	74	-	-
Van Tetering (2004) [7]	SF-36	312	NR	NR (25–64)	Exclusion of open fractures and age <25 and >65 years. Possible exclusion of patients who received secondary subtalar arthrodesis	ORIF or non-operative	23 (12–24)	67 (SD 27)	54 (SD 43)	57 (SD 26)	69 (SD 20)	60 (SD 21)	73 (SD 27)	72 (SD 38)	72 (SD 19)	-	-
Griffin (2014) [56]	SF-36	116	Sanders	47 (18–80)	Exclusion of open fractures, gross deformity, other serious leg injury or peripheral vascular disease	Operative (*n =* 62), non-operative (*n =* 54)	24 (NR)	-	-	-	-	-	-	-	-	40^c^ (SD 13)	54^c^ (SD 12)
Buckley (2014) [53]	SF-36 version 2	24	Sanders	40 (SD 13)	Closed Sanders type IV fractures; exclusion age <16 and >59 years, inability of diminishing smoking after injury	ORIF (*n =* 11), ORIF and primary arthrodesis (*n =* 13)	≥24 (24–84)	-	-	-	-	-	-	-	-	34^c^ (SD 11)	-
Heffernan (2000) [33]	SF-36	25	NR	44 (22–65) at time of injury	-	ORIF	30 (12–48)	Median83 (range 70–100)	-	Median84 (range 27–100)	-	-	-	-	-	-	-
Westphal (2004) [8]	SF-36	71	Sanders	42 (22–73) at time of injury	Exclusion of bilateral or open fractures, or having other injuries	ORIF, secondary arthrodesis (*n =* 4)	32 (11–90)	61 (SD 25)	52 (SD 43)	49 (SD 27)	58 (SD 20)	56 (SD 20)	79 (SD 23)	74 (SD 40)	69 (SD 21)	-	-
Brunner (2012) [46]	SF-36	54	OTA	NR	Exclusion of other fixation systems	ORIF	42 (24–82)	62	68	53	43	49	83	90	41	-	-
Kinner (2010) [41]	SF-36	44	Sanders	46 (18–65)	Complete clinical and radiographic documentation. Exclusion aged <65.	ORIF (*n =* 32), percutaneous (*n =* 12)	42 (12–60)	66	66	58	64	60	81	79	76	-	-
Beltran (2012) [45]	SF-36	15	Sanders	43 (16–67)	High-grade open calcaneal fractures only	Percutaneous	49 (13–106)	40 (SD 13)	43 (SD 16)	48 (SD 10)	52 (SD 8)	48 (SD 10)	44 (SD 10)	-	-	44^c^ (SD 12)	49^c^ (SD 13)
De Boer (2014) [55]	SF-36	78	Sanders/ Essex-Lopresti	46 (30–55) at time of injury	Exclusion of age >70, primary arthrodesis or amputation, Gustilo grade III, intoxication abuses or wheelchair-bound.	ORIF (*n =* 27), non-operative (*n =* 18), percutaneous (*n =* 33)	76 (54–88)	-	-	-	-	-	-	-	-	Median 48^a^	Median 57^a^
De Groot (2013) [48]	SF-36	41	NR	46 (SD 11) (21–66)	Exclusion of open fractures, severe mental illness or do not reside nearby	ORIF (*n =* 39), unknown (*n =* 2)	78 (24–192)	74 (SD 21)	65 (SD 41)	71 (SD 22)	70 (SD 19)	70 (SD 20)	85 (SD 22)	81 (SD 35)	79 (SD 20)	-	-
Makki (2010) [6]	SF-36 version 2	41	Sanders	56 (27–85) at time of follow-up	Exclusion of comminution of sustentacular fragment; open fractures; exclusion of secondary arthrodesis	ORIF	120 (84–180)	-	-	-	-	-	-	-	-	50^bc^ (SD 7)	54^bc^ (SD 4)
Sanders (2014) [58]	SF-36 version 2	66	Sanders/Essex-Lopresti	60 (SD 15) (29–91) at time of follow-up	Exclusion of open fractures, concomitant injuries, incomplete radiographic documentation, Sanders type IV, unsuited for operative treatment or subtalar fusion	ORIF	181 (126–256)	-	-	-	-	-	-	-	-	46^c^ (SD 9)	55^c^ (SD 11)

*ORIF* open reduction, internal fixation, *NR* not reported, *SD* = standard deviation

^a^Norm based scoring weighed upon the United States general population (1998) [71]; ^b^Norm based scoring: mean 50 (SD 10); ^c^Not reported how it was calculated

##### Patient characteristics

All studies, except two, had a number of patients which ranged from 15 to 78 [7, 56]. The number of patients in the study of van Tetering et al. [7] and Griffin et al. [56] was 312 and 116 patients respectively.

Mean age of the patients in the studies ranged from 36 to 60 years. Van Tetering et al. [7] only provided the range of age (25 to 64 years), and Brunner et al. [46] did not present any data about the age of the patients in their study. All studies evaluated operatively treated.

Longer follow-up time, Makki et al. patients; the studies of Van Tetering et al. [7], Griffin et al. [56] and de Boer et al. [55] also included non-operatively treated patients.

##### Fracture characteristics

Ten studies reported the classification of the type of calcaneal fracture, nine according to the Sanders classification, of which two also presented the Essex-Lopresti classification [6, 8, 38, 41, 45, 53, 55, 56, 58]. One study used the Orthopaedic Trauma Association (OTA) classification [46]. Three studies also reported the associated injuries of the studied population [41, 47, 55].

##### Follow-up time

In eleven studies the mean follow-up time ranged from 12 to 78 months [7, 8, 33, 38, 41, 45, 46, 48, 53, 55, 56]. Two studies had a substantial (2004) had a mean follow-up time of 120 months and Sanders et al. [58] 181 months [6].

##### HRQoL

Ten studies used the SF-36 and three studies used the updated version, SF-36v2. Moreover, the studies of Griffin et al. [56] and De Boer et al. [55] also reported the mean EQ-5D index score. Six studies presented the SF-36 results for all the subdomains [7, 8, 38, 41, 46, 48]. In one study the results are presented graphically; the numerical values were obtained from this graph [41]. In seven studies we have calculated the mean scores from the results in the study [6, 7, 38, 41, 45, 48, 58].

In the majority of the studies the SF-36 subdomain scores were similar. Several studies showed to be outliers in some subdomains. In the subdomain ‘physical functioning’ Heffernan et al. [33] and Beltran et al. [45] scored respectively the highest (83) and the lowest (40) score. The ‘bodily pain’ score was remarkably higher for Heffernan et al. [33] and De Groot et al. [48] which measured mean scores of respectively 84 and 71. Beltran et al. [45] showed to have a lower score of 43 in ‘social functioning’. The mean score of Brunner et al. [46] scored exceptionally lower in ‘mental health’ with a mean score of 41.

Beltran et al. [45] had the lowest scores in almost all the subdomains, in contrast to the study of Heffernan et al. [33] which had the highest scores in their reported subdomains ‘physical functioning’ and ‘bodily pain’. Brunner et al. [46] showed relatively low scores in the subdomains ‘general health’, ‘vitality’, and ‘mental health’, while the score in the subdomain ‘role-emotional’ was the highest of all studies.

The PCS and MCS are reported in six studies, of which only De Boer et al. [55] reported which reference population they used for calculating these scores [6, 45, 53, 56, 58]. Buckley et al. [53] reported just the PCS.

The mean EQ-5D index score was 0.69 in Griffin et al. [56], and the median EQ-5D index score in the study of De Boer et al. [55] was 0.78. The study of Griffin et al. [56] reported SF-36 MCS, SF-36 PCS and EQ-5D index score before and after trauma. The EQ-5D index showed a clinically significant decrease of the score with 0.21 points in patients with a DIACF. Also, the PCS and the MCS from the SF-36 survey decreased with respectively 11.9 and 2.0 points.

In one study, patients with DIACF are matched with a subject from the general population [8]. They are matched by age, gender, social state, comorbidity and place of residence. The results showed a statistically significant lower score in each subdomain for patients with DIACFs [8].

The data of the studies were not pooled because of a lack of homogeneity between studies; the studies have a great disparity in patient characteristics, type of fracture, treatment, and follow-up time [31].

Table 4 contains an overview of the subdomain scores, the studies are presented in order of follow-up time. The development of the scores through the years is graphically presented in the Appendix 1. The subdomain scores of ‘bodily pain’, ‘general health’, ‘vitality’, and ‘role-physical’ remained somewhat equal over time. The score of the ‘social functioning’, ‘mental health’, ‘role-emotional’, and ‘physical functioning’ increased over time.

### Discussion

A DIACF is an injury known to cause impairments, which have a significant impact on a person’s HRQoL, and on society and public health costs in terms of personal suffering and monetary losses.

Prior to this review we have observed numerous studies which evaluate the HRQoL. These studies tend to use different inclusion criteria and in particular observe specific patient groups. This might lead to skewed results and a misleading underestimation of the impact of a DIACF on the HRQoL. This in turn could limit the urge for improvements in the management of these fractures. We have performed this systematic review and aimed to evaluate the current literature on HRQoL in adult patients after sustaining a DIACF. This is the first systematic review that focuses solely on HRQoL after a DIACF.

This systematic review demonstrates that a lot of the studies have an increased risk of bias, mainly caused by the high lost to follow-up, selection bias and incomplete data reporting of patient and fracture characteristics. Moreover, this review shows that the SF-36 subdomains and the EQ-5D index scores are substantially lower in comparison to the period before the trauma and to the general population [46, 56, 59–63]. The results further present that the HRQoL after a DIACF is in particular affected in the subdomains related to the physical domain.

Several studies demonstrated scores in the SF-36 subdomains which were out of the range of the scores of the majority of the articles [33, 45, 46, 48]. The lower scores of Beltran et al. [45] can be explained by the inclusion of only patients with high-grade open fractures. These patients have a more severe injury and are more likely to have worse outcomes. We were not able to clarify why the scores of the other studies deviated. Possible other factors, besides the DIACF, might have influenced the HRQoL, for instance socio-economic status or severity of fracture [33, 46, 48]. These factors were not always reported in these studies.

The designation of these studies is very challenging; the focus in these studies should be on decreasing the risk of bias. The following suggestions might decrease the risk of bias and benefit future research. Patients who sustained a DIACF are considered to be a group who are difficult to follow-up. Nonetheless, all the studies with a medium risk of bias have a decent follow-up of 75 % or better. To increase the follow-up rate in future studies; it may be worthwhile to use a shorter questionnaire (e.g. EQ-5D). In addition, we recommend that patient and fracture characteristics of the eligible population are reported to interpret the bias caused by loss to follow-up.

Furthermore, we suggest that future studies include data regarding factors that influence HRQoL, such as socio-economic status, acceptability of the disease in the population, and the quality of health care [60, 64, 65]. Also data of certain patient and injury characteristics, e.g. age, concomitant injuries, and classification of calcaneal fracture, are important to report because these factors influence HRQoL substantially [28]. Adding this information in these studies increases the generalizability and applicability to other study populations and the individual patient.

We have demonstrated in the critical appraisal (Table 3) that the majority of the studies had a selection bias. These studies excluded patients with open or bilateral fractures, multiple injured patients, patients who received secondary subtalar arthrodesis (‘failure’ of treatment), and patients who were not eligible for a certain treatment (Table 3). It is likely that these patients have worse HRQoL outcome compared to patients with an isolated and closed DIACF. Therefore, the results may be distorted and the impact on the HRQoL might be even worse than presented in this review. Thus, ideally, future studies include all type of patients and fractures, and all their characteristics should be reported as discussed above.

The development of the subdomain scores over time (Table 4 and Appendix 1) shows that ‘social functioning’, ‘physical functioning’, ‘mental health’, and ‘role-emotional’ improve over time. This could indicate that the rehabilitation period for this injury is a very time-consuming process or that patients learn to cope with their limitations. Given the changes in HRQoL over time, we might suggest that it is important to measure HRQoL during a long follow-up period.

Several studies in our review only reported the SF-36 PCS and MCS results. These scores are useful to summarize the HRQoL. Though, some studies demonstrated that these summarized scores possibly do not reflect accurately the HRQoL in comparison to the scores per subdomain [66, 67]. Moreover, an advantage of the scores per subdomain is the possibility to compare the survey with other surveys, such as the Maryland Foot Score, AOFAS Ankle Hindfoot Scale, and Iowa Calcaneal Score or to combine the result in a meta-analysis [8, 27, 33, 52, 67–70].

The significantly decrease in HRQoL indicates that a DIACF is a life-changing event, regardless of HRQoL status before the DIACF, type of fracture, non-operative or operative treatment, or additional injuries. It has a great impact on a person’s physical and social function.

The considerable loss in HRQoL shows the need for advancements in the management strategy of a DIACF in order to improve functional outcome. In current literature there is no consensus yet what the best treatment of these patients should be. A recently published randomized controlled trial demonstrated that there actually is no difference in subjective and objective outcomes after two years between non-operative and operative treatment of DIACF. [56] Although operative treatment is considered the gold standard nowadays in treatment of DIACF, this study suggests that patients who were operatively treated could still endure a severe loss in their HRQoL. Apart from the treatment of a fracture, other management strategies may be introduced. Physical functioning might be improved by early involvement of the rehabilitation physician, and psychotherapy might be helpful to cope with potentially impaired mental functioning after a trauma that caused a DIACF.

In conclusion, this systematic review indicates that DIACF is a life-changing event for most patients. However, we should be careful to deduct definite conclusions; we revealed that the identified studies have a medium to high risk of bias that might cause underestimation of the HRQoL after a DIACF. Thus, it is challenging to make reliable and valid conclusions.

In future, we recommend that research aims to decrease risk of bias. Ideally, future studies on HRQoL should use shorter questionnaires to aim for a higher follow-up rate, present data on the patients which were not included, and preferably include all patients with DIACF despite their characteristics which likely influence the HRQoL outcome negatively. Furthermore, it would be useful to present all relevant patient characteristics and injury characteristics. All of this is necessary to lower the risk of bias as best as possible in this challenging population; in order to interpret the results better and to create a more representative, inclusive image on the consequences on the daily lives of patients who endured a DIACF.

## Conclusions

- This systematic review reveals that the identified studies have a medium to high risk of bias that might cause underestimation of the HRQoL after a DIACF; hence, it is challenging to make reliable and valid conclusions.
- This systematic review indicates that DIACF is a life-changing event for most patients.

## Figures and Tables

**Fig. 2:**
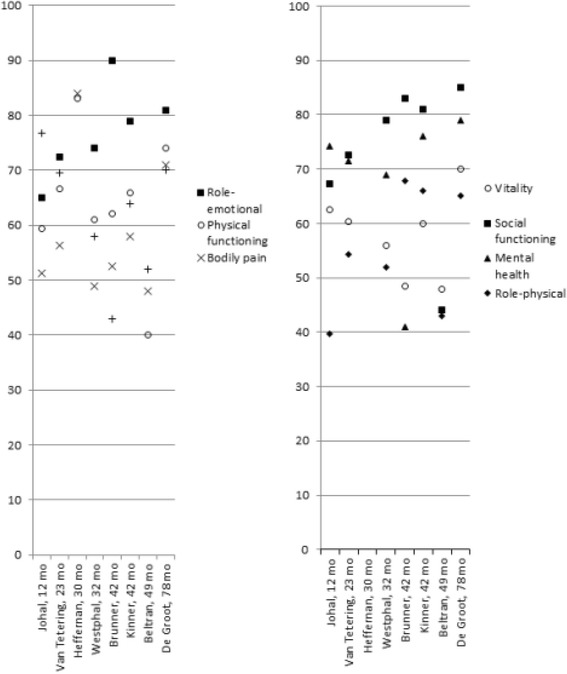
A The SF-36 subdomain scores development in time

## Abbreviations

ACG: Amy Gunning
DIACF: Displaced intra-articular calcaneal fracture
EQ-5D: EuroQol 5D
EQ-6D: EuroQol 6D
GA: Georgios Alexandridis
HRQoL: Health-related quality of life
LPHL: Luke Leenen
MCS: Mental component summary
OTA: Orthopaedic Trauma Association
PCS: Physical component summary
PRISMA: Preferred Reporting Items for Systematic Reviews and Meta-Analyses
SF-36: Medical Outcomes Study 36-item Short Form Health Survey
SF-36v2: Medical Outcomes Study 36-item Short Form Health Survey version 2.0
